# Sexually dimorphic responses in androgen metabolism and signalling in the non-human primate placenta to moderate maternal undernutrition

**DOI:** 10.1186/s13293-025-00771-y

**Published:** 2025-11-07

**Authors:** Ashley S. Meakin, Peter W. Nathanielsz, Cun Li, Vicki L. Clifton, Hillary F. Huber, Michael D. Wiese, Janna L. Morrison

**Affiliations:** 1 Early Origins of Adult Health Research Group, Health and Biomedical Innovation, UniSA: Clinical and Health Sciences, University of South Australia, GPO Box 2471, Adelaide, SA 5001 Australia; 2 Department of Animal Science, University of Wyoming, Laramie, WY USA; 3 Mater Medical Research Institute, The University of Queensland, Brisbane, QLD Australia; 4 Southwest National Primate Research Center, Texas Biomedical Research Institute, San Antonia, TX USA; 5 Centre for Pharmaceutical Innovation, Clinical & Health Sciences, University of South Australia, Adelaide, SA Australia

**Keywords:** Androgen signalling, Androgen receptor, Baboon, Placenta, Cytochrome P450, Sex differences, Developmental programming, Nonhuman primate

## Abstract

**Background:**

Maternal nutrient restriction (MNR) can increase maternal androgen concentrations during pregnancy and cause placental dysfunction leading to reduced fetal growth, especially in males. Placental androgen metabolism, as well as differential expression and subcellular localisation of androgen receptor (AR) variants, modulates androgen signalling, which may benefit placental function; however, the impact of MNR on these adaptations remains undefined. We characterised the impact of MNR and fetal sex on placental androgen signalling in a non-human primate model of pregnancy.

**Methods:**

Pregnant baboons (*Papio* spp.) were randomly assigned to control diet (Ctrl; offspring female *n* = 5, male *n* = 6) or MNR diet (70% of global Ctrl; offspring female *n* = 5, male *n* = 5) at 0.16 gestation (term = ~ 180 days). Fetuses were delivered by Caesarean section at 0.9 gestation and placenta collected. Molecular measures of sex steroid signalling and placental function were quantified using established LC-MS/MS assays, Western blot, and qRT-PCR. Data were analysed using two-way ANOVA (factors: diet, sex) with Tukey’s multiple comparison test.

**Results:**

*CYP17A1*, *SRD5A1*, and *PGF* expression was higher, whereas *HSD3B1*, *CYP19A1*, and *ANGPT2* was lower in male compared to female placentae, independent of diet. *KDR* expression and CYP19A1 activity increased in MNR versus Ctrl in females only. Cytoplasmic expression of the antagonistic AR variant, AR-45, was higher in males, whereas MNR increased cytoplasmic and nuclear AR-45 expression independent of sex.

**Conclusions:**

Differences in placental steroidogenic and angiogenic genes, as well as androgen metabolism and signalling, may explain sex-specific placental responses to MNR. Better understanding of molecular regulators of androgen signalling may lead to novel, targetable therapeutics that improve placental function in complicated pregnancies associated with increased androgen concentrations.

## Abstract (plain language summary)

The placenta is a transient organ that plays several key biological roles throughout pregnancy, all of which are needed to ensure fetal growth and survival. Sex differences in placental function are known. Male placentae generally prioritise growth pathways at the expense of adaptation to any current pregnancy stressors compared with female placentae. Pregnancies that are complicated by maternal undernutrition (MNR) are associated with sex-specific fetal growth and survival outcomes, with male fetuses displaying poorer outcomes than females. The underlying mechanisms that contribute to these sex-specific outcomes are not known but may partly be explained by changes to placental androgen signalling, a pathway that if dysregulated can contribute to impaired placental function and poor fetal outcomes. Therefore, in this study we characterised the effect of sex and/or MNR on placental androgen signalling in the non-human primate (baboon) placenta. Independent of maternal diet, female placentae had higher gene expression of enzymes and other factors that support placental function, whereas male placentae had higher gene expression of enzymes involved in androgen production. Intriguingly, in response to MNR, female placentae increased androgen metabolism whereas male placentae suppressed this response. Our study indicates sex differences in placental androgen signalling may contribute to poorer outcomes for males in response to pregnancy stressors.

## Highlights

- Pregnancies complicated by maternal nutrient restriction (MNR) are associated with sex-specific fetal growth and survival outcomes, with males generally displaying poorer outcomes.
- The mechanisms that contribute to these sex-specific outcomes may be partly regulated by changes to placental androgen signalling, a pathway mediated by the full-length androgen receptor (AR-FL). Therefore, advanced understanding of this pathway is needed to improve placental function and reduce the prevalence of associated risks.
- Independent of maternal diet, female placentae had higher expression of *CYP19A1*, *HSD3B1*, and *ANGPT2*, whereas male placentae had higher *CYP17A1*, *SRD5A1*, *PGF* expression, and greater cytoplasmic sequestration of the antagonistic androgen receptor (AR) isoform, AR-45.
- In response to MNR, female placentae had higher activity of enzymes involved in testosterone metabolism, as well as markers of angiogenesis, whereas male placentae suppressed testosterone metabolism.
- Sex differences in placental androgen signalling pathways may contribute to poorer outcomes for males in response to pregnancy stressors.

## Background

The placenta is a transient, endocrine organ that plays an essential role in maintaining pregnancy health. While multifaceted, the placenta’s primary function is to establish a network of vasculature that necessitates oxygen and nutrient transfer from mother to fetus, thereby ensuring fetal growth and development is maintained [6]. Stressors during pregnancy, including those associated with maternal undernutrition, can impact placental function at the molecular and structural level, which consequently affects fetal growth and development [5, 8]. Intriguingly, sex differences in the placental response to maternal undernutrition have been observed, with males displaying impaired adaptations compared with females [32, 45]. These data support the ‘male disadvantage hypothesis’, which states that males prioritise growth pathways at the expense of placental reserve capacity, thereby increasing the risk of intrauterine morbidity and mortality [14]. In contrast, female placentae appear responsive to shifts in the intrauterine environment resulting in reduced prevalence of adverse outcomes [37]. The underlying mechanisms that contribute to these sex differences in response to maternal stressors, including those associated with undernutrition, remain unclear but likely involve changes to placental sex steroid signalling pathways.

Sex steroid signalling, which consists of sex hormone (e.g., androgens, estrogens) biosynthesis and metabolism, steroid receptor (e.g., androgen receptor (AR), estrogen receptor (ER)) protein variant expression, and AR- or ER-mediated transcriptional regulation of target genes, may be such pathways involved in the sexual dimorphism of placental adaptations to maternal undernutrition [31]. In female-bearing bovine pregnancies, undernutrition in the first half of gestation has been shown to increase circulating maternal concentrations of the androgen testosterone, with no impact on fetal or placental weight at delivery [43]. Less is known about the circulating androgen profile in male-bearing pregnancies complicated by maternal undernutrition; however, in response to maternal caloric restriction, male murine placentae had increased expression of several genes involved in androgen biosynthesis [32]. Although androgens are essential for normal fetal growth and development, particularly for males [62], chronic activation of placental androgen signalling has been associated with impaired angiogenesis and vasculogenesis, and thus function [4, 19, 48]. Importantly, the placenta is a rich source of androgen metabolising enzymes and has distinct expression profiles of AR protein variants. These variants can function to modulate androgen signalling and thus mitigate placental dysfunction induced by excess androgen signalling [37].

Alternative splicing of the *AR* gene gives rise to multiple transcript variants, some of which function as endogenous modulators of the androgen signalling axis [35]. Specifically, the N-terminally truncated variant AR-45 antagonises the full-length AR (AR-FL) by forming a proposed non-responsive heterodimer [1]. AR-45 expression is high in the placenta when compared with other organs [30] and may therefore serve as an integral regulator of androgen signalling under physiological conditions. Indeed, previous work in humans reported males, but not females, were more likely to be small for gestational age when AR-45 expression was reduced in the placenta [36]. The same study also reported in vitro that high androgen concentrations reduced AR-45 expression but not AR-FL. More recently in baboons, AR-45 expression in the liver of male fetuses exposed to maternal overnutrition (a stressor that is associated with increased androgens) was reduced [40]. Whether or not similar sex-specific adaptations exist in placentae from pregnancies complicated by maternal undernutrition is not known. However, it is speculated that the male fetoplacental unit prioritises androgen signalling pathways, independent of the presence or absence of a pregnancy stressor, at the expense of placental function and thus fetal survival [8, 37].

Metabolism of androgens to estrogens via aromatase (cytochrome P450 (CYP) 19A1) is considered a key mechanism by which the placenta protects the fetoplacental unit from increasing androgen concentrations. Previous work in humans reported increased aromatase gene and protein expression in female placentae from preeclamptic pregnancies when compared with males [49], which the authors concluded may be a sexually dimorphic response to protect the female fetus from virilization associated with increased maternal androgen concentrations associated with preeclampsia. This increase in aromatase may also benefit the female placenta in response to a suboptimal intrauterine environment. Estrogen signalling via ERα and ERβ – the two main ER isoforms – is involved in trophoblast viability, proliferation, and invasion; extracellular matrix remodelling; nitric oxide (NO) and vascular endothelial growth factor (VEGF) synthesis; and vasodilation of uterine, placental, and umbilical arteries [2]. There is a balance between androgen and estrogen signalling, and changes to the intrauterine environment that tip this balance to a more androgenic environment may compromise placental function and thus fetal growth and survival outcomes.

Considering maternal undernutrition induces sex differences in fetoplacental adaptations and is known to alter the maternal hormonal milieu by increasing circulating androgen concentrations, in the current study, we aimed to comprehensively characterise placental-specific androgen and estrogen signalling axes using an established non-human primate model of maternal undernutrition.

## Methods

All animal procedures were approved by the Texas Biomedical Research Institute Institutional Animal Care and Use Committee (IACUC) and conducted in an Association for Assessment and Accreditation of Laboratory Animal Care (AAALAC) International-certified facility. The experimental design was informed by the ARRIVE guidelines [24] and the 3Rs (https://www.nhmrc.gov.au/research-policy/ethics/animal-ethics/3rs).

### Animal model

Baboons (*Papio* spp.) are the closest translational model for human pregnancy due to their similarity in genetics, placental structure and function, body size, and reproductive physiology [3, 9, 10]. Baboons were housed in outdoor group cages with normal physical and social interaction. The custom-designed housing system allowed for individual control and monitoring of food intake [50]. After confirmation of pregnancy at 0.16 gestation, baboons were randomly assigned to either a control diet (Ctrl) or a maternal nutrient restricted (MNR) diet. *Ad libitum* food intake (Purina Monkey Diet 5LEO, Purina, St Louis, MO, USA) of Ctrl mothers was calculated weekly on a per kilogram basis. The MNR group received 70% of the average daily *ad libitum* amount eaten on a weight-adjusted basis by Ctrl animals of the same gestational age. The nutritional content of the diet included 14% calories by fat, 0.22% glucose, 0.24% fructose, and metabolizable energy content of 2.98 kcal/g.

### Caesarean sections and tissue collection

Pregnant baboons underwent Caesarean section at 0.9 gestation (term ~ 180 days) using standard sterile surgical techniques [51]. Briefly, baboons were pre-medicated with ketamine hydrochloride (7–10 mg/kg intramuscularly) and maintained at a surgical plane of anaesthesia with isoflurane (1–2% inhaled). After hysterectomy, the fetus was exsanguinated under general anaesthesia as approved by the American Veterinary Medical Association Panel on Euthanasia [46]. Morphometric measurements were collected, and placental tissue samples obtained immediately, snap frozen in liquid nitrogen and stored at −80 °C. Buprenorphine hydrochloride (0.015 mg/kg/day, 2 daily doses for 3 days) was administered for postoperative maternal analgesia [51]. After recovery from anaesthesia, baboons were individually caged for the initial post-operative period and then group-housed for 90 days with a vasectomised male to prevent pregnancy before the surgical site was completely healed.

### Microsome extraction

Placental microsomes were extracted using differential centrifugation as previously described [33, 39, 54]. Briefly, frozen tissue samples of ~ 250 mg were homogenised (Tissue Lyser, Qiagen, Switzerland) in 600 µl of homogenising buffer (1.15% KCl, 1mM EDTA, pH 7.4). The homogenate was centrifuged at 9,000 *g* for 20 min at 4 °C. The supernatant was then transferred and centrifuged at 16,000 *g* for 60 min at 4 °C. Supernatant was then discarded and the microsomal pellet was resuspended in buffer (100mM potassium phosphate buffer, 20% glycerol, pH 7.4). The amount of protein in each extraction was determined using a Micro Bicinchoninic Acid (BCA) Protein Assay Kit (Pierce, Thermo Fisher Scientific Inc, Rockford, USA) with bovine serum albumin (2 mg/ml) to generate a standard curve. Microsomal extractions were stored at −80 °C until enzymatic assays.

### CYP activity assay

To quantify the activity of testosterone metabolising enzymes, the rate of testosterone to 2α-hydroxytestosterone (CYP3A7), 6β-hydroxytestosterone (CYP3A4/5/7), estradiol (CYP19A1), or dihydrotestosterone (SRD5A1) was determined by adding 350 µM testosterone to 70 µg of microsomal protein, 10 mM NADPH, and assay buffer (50 mM phosphate buffer, 2 mM magnesium chloride, pH 7.4) to a total reaction volume of 100 µl [40]. Reactions were stopped by adding 100 µl methanol containing 100 ng/ml 6β-hydroxytestosterone-d3 (Toronto Research Chemicals, Canada). Incubation mixtures were centrifuged at 12,000 *g* for 10 min at 4 °C and the supernatant transferred to a liquid chromatography tandem mass spectrometry (LC-MS/MS) vial for subsequent analysis.

### Liquid chromatography tandem mass spectrometry (LC-MS/MS)

Quantitation of testosterone metabolites was determined using LC-MS/MS (SCIEX 6500 Triple-Quad (SCIEX, US) with Shimadzu Nexera XR (Shimadzu, Japan)), as previously described [40]. Briefly, incubation mixtures were transferred to a 0.4 ml autosampler vial and then 10 µl of sample was injected onto an ACQUITY BEH C18 Column (130 Å, 1.7 μm, 2.1 mm × 100 mm (Waters Corp., US)). Mobile phases were 0.2% formic acid in water (A) and 0.2% formic acid in 100% acetonitrile (B). Flow rate was 0.2 ml/min and mobile phase B was initially 15% for 1 min, increased to 40% over 1.5 min, then to 50% over 6 min, and then to 98% over 3.5 min. The gradient was held at 98% for 0.8 min, after which it returned to 15% over 0.2 min held at 15% for 2 min prior to injection of the next sample.

### Quantification of placental subcellular steroid receptor expression

Tissue subcellular fractionation and western blotting was performed as previously described [36]. Subcellular lysates (50 µg) were electrophoresed via SDS-PAGE and then transferred onto a polyvinylidene fluoride (PVDF) membrane (Hybond ECL, GE Healthcare, Australia). Membranes were blocked in 5% BSA in TBS with 1% Tween (TBS-T) for 1 h at room temperature, and then incubated with affinity purified polyclonal rabbit anti-AR (1:1000, Cell Signalling, USA Cat no. 54653; 1:100, Abcam, UK Cat no. ab74272), affinity purified polyclonal rabbit anti-ERα (1:1000, Thermo Scientific, USA Cat no. PA1309), or affinity purified polyclonal rabbit anti-Erβ (1:1000, Thermo Scientific, USA Cat no. PA1310B) antibodies. The appropriate secondary antibody (goat anti-rabbit; 1:20000) was applied for 1 h. Membranes were subsequently probed with anti-β actin (1:4000, Bethyl laboratories, USA Cat no. A300-491 A) as loading control. SuperSignal West Pico Chemiluminescent Substrate (Thermo Scientific) was used to detect reactive bands by enhanced chemiluminescence. Western blots were imaged using ImageQuant LAS 4000 (GE Healthcare) and protein abundance was determined by densitometry using Image Quant software (GE Healthcare) and initially normalised to their respective loading control. As per previously published work examining steroid receptor isoform protein data [11, 40], protein expression of each AR isoform was normalised to matched AR isoform expression in a pooled sample, which allowed for comparisons between membranes.

### Quantification of placental mRNA expression

All essential information regarding the qRT-PCR procedure is included as per the MIQE guidelines [7]. RNA was extracted from tissue samples (~ 50 mg) using QIAzol Lysis Reagent solution and RNeasy purification columns as per manufacturer guidelines (Qiagen, Germany). Total RNA was quantified by spectrophotometric measurement on a NanoDrop Lite (Thermo Fisher Scientific) and RNA integrity was assessed using agarose gel electrophoresis. cDNA was synthesized from 1 µg of total RNA using the Superscript III First Strand Synthesis System (Invitrogen, USA) as per manufacturer’s guidelines. Controls containing either no RNA transcript or no Superscript III were used to test for reagent contamination and genomic DNA contamination, respectively. The geNorm component of qbaseplus 3.4 software (Biogazelle, Belgium) was used to determine the most stable reference genes from a panel of candidate reference genes and the minimum number of reference genes required to calculate a stable normalisation factor, as previously described [29, 34, 53]. Three stable reference genes (YWHAZ, TBP and ACTB) were run in parallel with all target genes using Qiagen QuantiNova SYBR Green (Qiagen) on a Quantstudio 7 Pro Real-time PCR system (Applied Biosystems, USA). Target genes were chosen a priori to investigate key pathways involved in growth, nutrient transport, steroidogenesis, and angiogenesis (Table 1). The abundance of each transcript relative to the abundance of stable reference genes was calculated and expressed as mRNA mean normalised expression (MNE).

**Table 1:** Primer details

Gene	Forward primer	Reverse primer
*ANGPT2*	CCTACGGGAAGATAACGGATAAG	GTCAGATTGCAGTGGGAAGA
*CYP11A1*	AAGTCCACCTTCACCATGTC	ACTCCTATGGGTCTCTGGTAAT
*CYP17A1*	AGTATCCTAAGAGCCTCCTGTC	GAGAAGTCCTTGCCCTTCTTAAT
*CYP19A1*	GATACGGGCTGGACATCAAA	GTGATCCTTGGTGTCCTCTTAC
*FLT1*	CAGAGATCAGGAAGCACCATAC	GCAGTGATAGACACCTTCATCC
*HSD3B1*	GGCCAGAGATCAAAGGGATAAG	TCGCCAGGAAACAGAAGATG
*IGF1*	TCCTCAGACTCTTCTCCTTCC	TGCTACAACATGGGCTACAG
*IGF1R*	TGAAAGGAAGCGGAGAGATG	GTGAAAGGCCGAAGGTTAGA
*IGF2*	CTGGTGCTTCTCACCTTCTT	GGAAACAGACTCCTCAACGAT
*IGF2R*	TGGAACTTGGGACTGAGTAATG	CGGAAGTTGTAGGTGGAGTTATC
*KDR*	GTGGTCTCTCTGGTTGTGTATG	CCTCCACACTTCTCCATTCTTC
*PGF*	AGCCACTTCCCTCTCTTCT	CCACTCTGCATGTGTCTCTTAG
*RPLPO*	CTTGTCTGTGGAGACGGATTAC	TCTTCCTTGGCTTCAACCTTAG
*SCARB1*	GTTTCTCTCCCATCCTCACTTC	GTCCAATGCCTGCGATAGAT
*SLC2A1*	TCCTCATCGCCCAGGTATT	CAGCTTCTTCAGCACACTCTT
*SLC2A4*	CCTCAGAAGGTGATTGAACAGA	CCAAGCCACTGAGAGATGATAC
*SRD5A1*	TCCGGCCTAATTACATGCTTAC	CTAGACGTGGAAGGACCTTTG
*STAR*	CAACCCTAGCACGTGGATTAG	CACATCTGGGACCACTTTACTC
*TBP*	GCTTCAGAGAGTTCTGGGATTG	GTGGTTCGTGGCTCTCTTATC
*TGFB1*	GGAAACCCACAACGAAATCTATG	TGAGGTATCGCCAGGAATTG
*VEGFA*	CGGGTCAGATGGACAGAAAG	ACTCAGAAGCAAGTGAGAGTAAG
*YWHAZ*	TCACAAGCAGAGAGCAAAGT	CCCAGTCTGATAGGATGTGTTG

### Statistical analysis

All statistical analyses were performed using GraphPad Prism 8 (GraphPad Software, Inc., USA). Initial normality testing was conducted on data within each study group. For data where at least one of the groups was not normally distributed (Shapiro-Wilk P-value < 0.05), non-parametric tests including Mann-Whitney U test or Kruskal-Wallis with Dunn’s multiple comparisons post-hoc analysis were performed. For data where all groups were normally distributed (Shapiro-Wilk P-value > 0.05), two-way ANOVA (factors = diet and sex) with Tukey’s post-hoc analysis was performed. Potential outliers were detected using Grubbs’ test. Data are presented as mean ± SD, unless stated otherwise. The alpha level was 0.05.

## Results

### Cohort measures

At 0.9 gestation, maternal body weight (MW), fetal body weight (FW), placental weight (PW), FW: MW, and FW: PW did not differ by sex or diet (Table 2).

**Table 2:** Maternal, placental, and fetal weights

	Ctrl		MNR				
	Female (*n* = 5)	Male (*n* = 6)	Female (*n* = 5)	Male (*n* = 5)	*P*_diet_	*P*_sex_	*P*_intx_
Maternal body weight (MW, kg)	18.27 ± 0.52	19.05 ± 1.12	17.09 ± 1.77 (*n* = 4)	16.74 ± 1.51	0.198	0.874	0.667
Fetal body weight (FW, g)	776.50 ± 41.38	802.93 ± 40.90	696.92 ± 28.75 (*n* = 4)	760.54 ± 24.39	0.101	0.219	0.605
Placental weight (PW, g)	193.12 ± 16.19	213.91 ± 13.79 (*n* = 5)	183.44 ± 20.51 (*n* = 4)	186.19 ± 14.18	0.289	0.515	0.544
FW: MW	0.043 ± 0.001	0.043 ± 0.002	0.042 ± 0.003 (*n* = 4)	0.047 ± 0.003	0.479	0.323	0.292
FW: PW	4.123 ± 0.289	3.785 ± 0.235 (*n* = 5)	3.800 ± 0.317 (*n* = 4)	4.216 ± 0.365	0.867	0.904	0.253

Data presented as mean ± SD. Statistical analysis: two-way ANOVA with Tukey’s post-hoc analysis. Ctrl = control diet; MNR = maternal nutrient restriction; intx = interaction effect. *P* < 0.05 was considered statistically significant

### Impact of maternal diet on gene expression of placental growth factors and glucose transporters

**Fig. 1:**
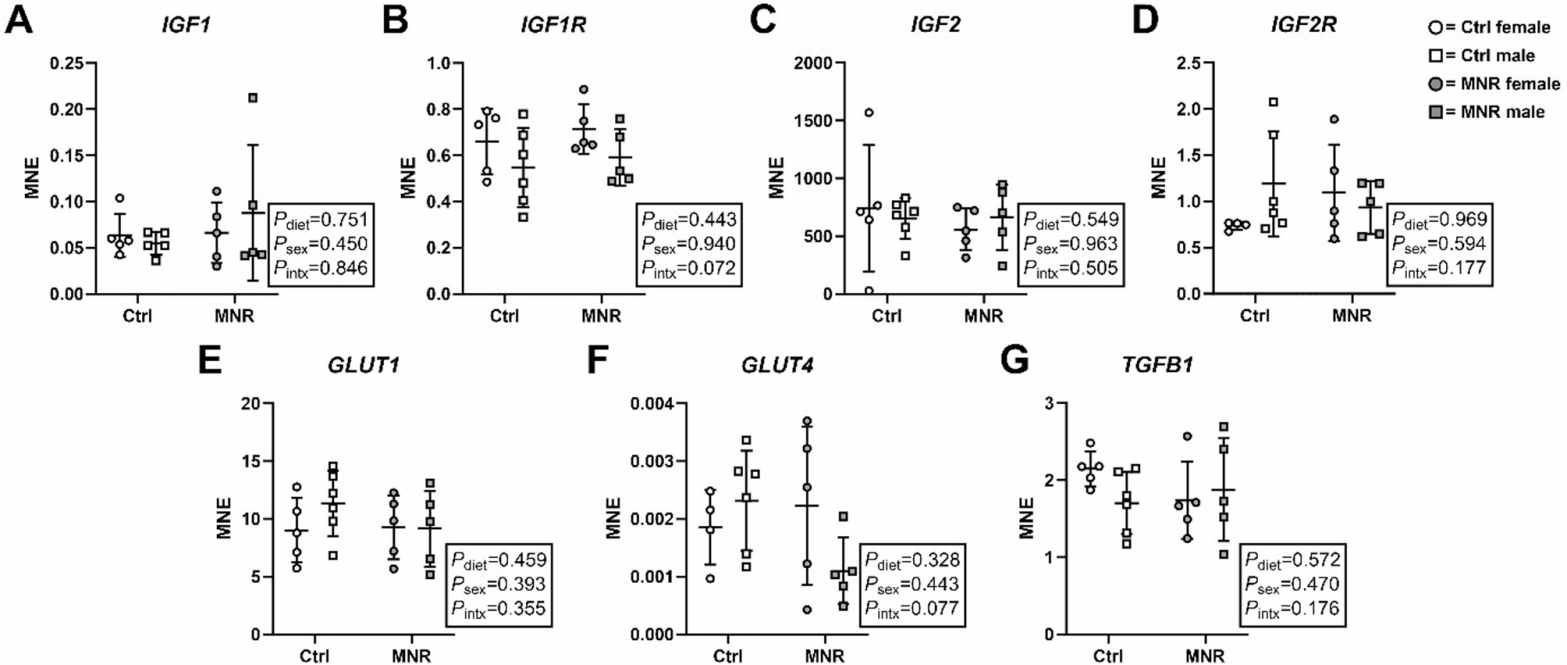
Expression of genes involved in growth and glucose transport is not impacted by diet or sex. mRNA expression of **(A)** *IGF1*, **(B)** *IGF1R*, **(C)** *IGF2*, **(D)** *IGF2R*, **(E)** *SLC2A1*, **(F)** *SLC2A4*, and **(G)** *TGFB1* was measured in female (circles) and male (squares) placenta samples from Control (Ctrl; open data points, female *n* = 5, male *n* = 6) and maternal nutrient restriction (MNR; closed data points, female *n* = 5, male *n* = 5) pregnancies. MNE = Mean normalised expression. Statistical analysis: two-way ANOVA (fixed factors: diet and sex; intx = interaction effect) with Tukey’s multiple comparison test. *P* < 0.05 was considered statistically significant. Data presented as mean ± SD

The mRNA expression of *IGF1*, *IGF1R*, *IGF2*, *IGF2R*, *SLC2A1*, *SLC2A4* and *TGFB1* did not differ by diet or sex (Fig. 1A-G).

### Sex and maternal diet impacts gene expression of placental angiogenic factors

**Fig. 2:**
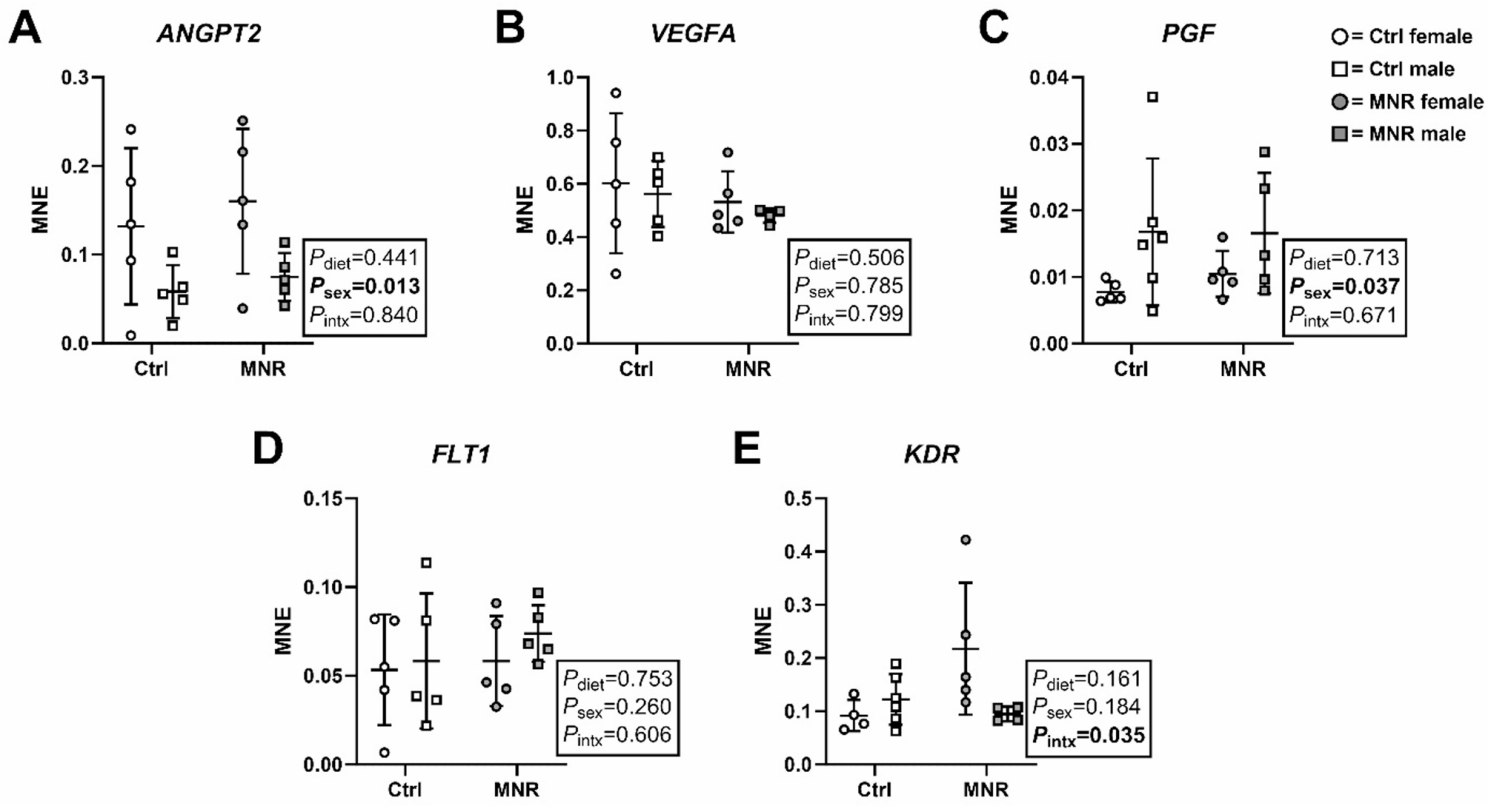
Expression of angiogenic transcripts is impacted by sex and maternal diet. Transcript expression of **(A)** *ANGPT2*, **(B)** *VEGFA*, **(C)** *PGF*, **(D)** *FLT1*, and **(E)** *KDR* was measured in female (circle data points) and male (square data points) placenta samples from Control (Ctrl; open data points, female *n* = 5, male *n* = 6) and maternal nutrient restriction (MNR; closed data points, female *n* = 5, male *n* = 5) pregnancies. MNE = Mean normalised expression. Statistical analysis: two-way ANOVA (fixed factors: diet and sex; intx = interaction effect) with Tukey’s multiple comparison test (*=*P* ≤ 0.05). *P* < 0.05 was considered statistically significant. Data presented as mean ± SD

*ANGPT2* expression was lower in males compared with females, independent of diet (sex effect [*F*_(1, 16)_ = 7.870, *P* = 0.013], Fig. 2A), whereas *PGF* expression was higher in males compared with females (sex effect [*F*_(1, 17)_ = 5.140, *P* = 0.037], Fig. 2C). *KDR* expression was higher in placentae from MNR compared with Ctrl pregnancies but only in females (interaction effect [*F*_(1, 15)_ = 5.347, *P* = 0.035, Fig. 2E). mRNA expression of *VEGFA* and *FLT1* did not differ by maternal diet or sex (Fig. 2B & D).

### Activity of testosterone metabolising enzymes is increased in female placentae from MNR pregnancies

**Fig. 3:**
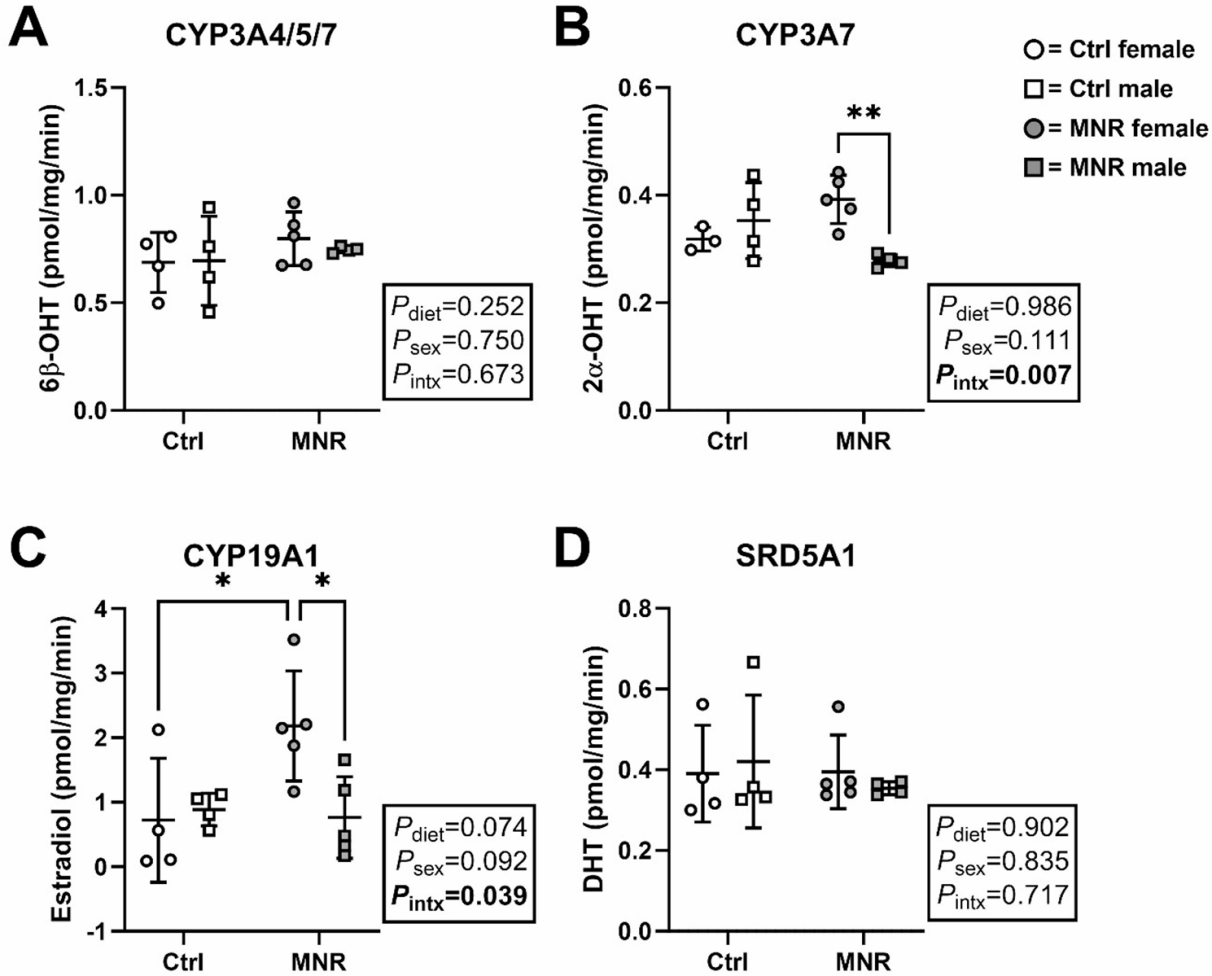
Testosterone metabolism in the baboon placenta is impacted by maternal diet in a sex-specific manner. Activity of placental-specific **(A)** CYP3A4/5/7, **(B)** CYP3A7, **(C)** CYP19A1 and **(D)** SRD5A1 was measured by quantifying the amount of metabolite produced per mg microsomal protein per minute of incubation (pmol/mg/min). Functional activity was measured in control (Ctrl; open data points, female *n* = 4, male *n* = 4) and maternal nutrient restriction (MNR; closed data points, female *n* = 5, male *n* = 5) pregnancies. Statistical analysis: two-way ANOVA (factors: diet and sex; Intx = interaction effect) with Tukey’s multiple comparison test (*=*P* ≤ 0.05, **=*P* ≤ 0.01). *P* < 0.05 was considered statistically significant. Data presented as mean ± SD

Female placentae from MNR pregnancies had higher conversion rates of testosterone to 2α-OHT when compared with MNR males (interaction effect [*F*_(1, 12)_ = 10.46, *P* = 0.007], Fig. 3B). An interaction effect was observed for the conversion rate of testosterone to estradiol [*F*_(1, 14)_ = 5.218, *P* = 0.039], with rates higher in female placentae from MNR pregnancies when compared with Ctrl female and male MNR placentae (Fig. 3C). Conversion rates of testosterone to DHT (SRD5A1) or 6β-OHT (CYP3A4/5/7) did not differ between sex or diet (Fig. 3A & D).

### Expression of placental AR protein variants is impacted by maternal diet or sex

**Fig. 4:**
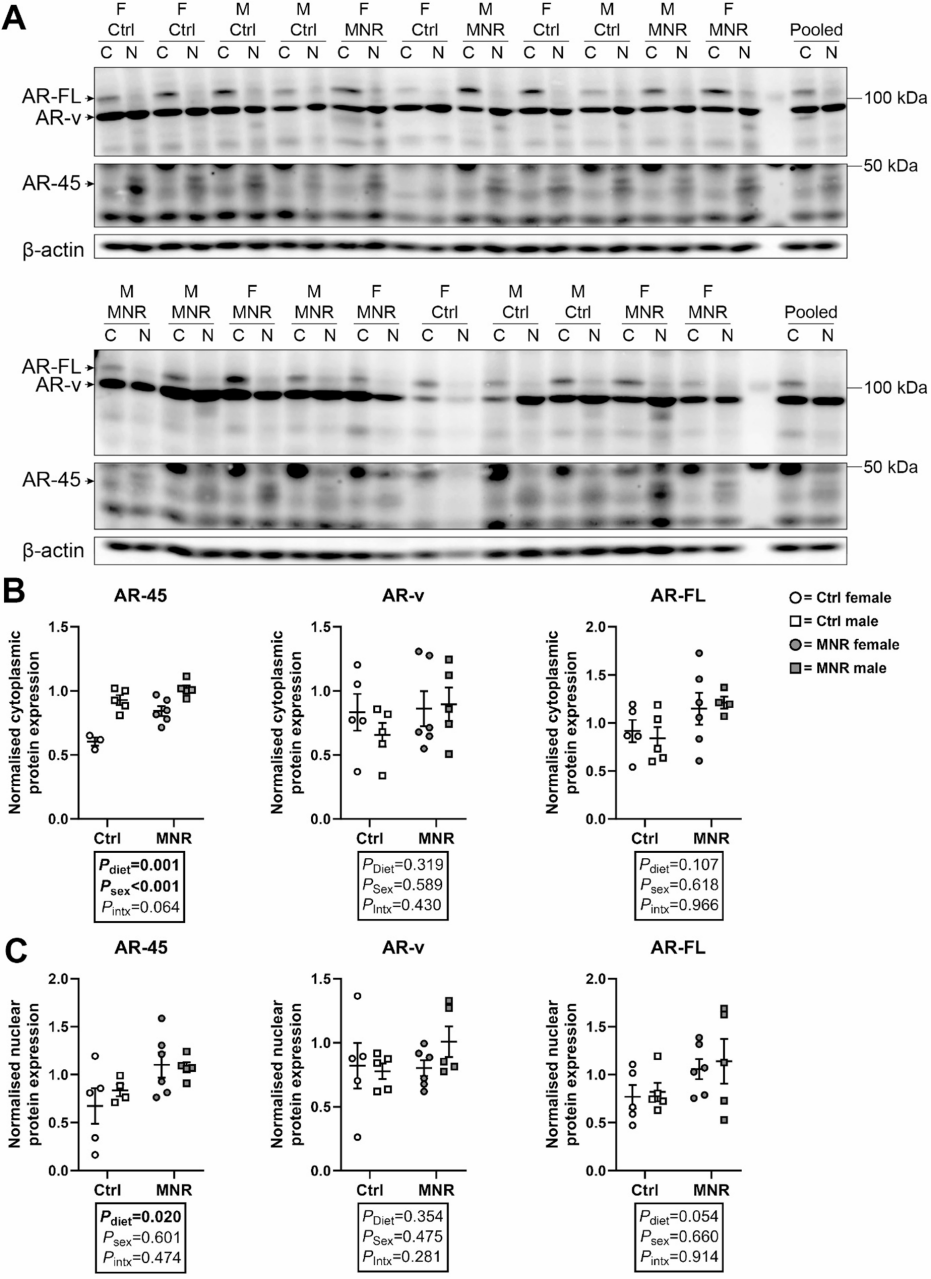
Androgen receptor (AR) protein variant expression in the baboon placenta is impacted by maternal diet and sex. **(A)** Cytoplasmic (C) and nuclear (N) protein extracts of female (F) and male (M) placenta samples from control (Ctrl) and maternal nutrient restriction (MNR) pregnancies. AR-FL, AR-v, and AR-45 were detected in all samples. Membranes were probed with β-actin as a loading control. **(B)** Cytoplasmic and **(C)** nuclear expression of AR-45, AR-FL, and AR-v in female (circles) and male (square) placentae of Ctrl (open data points, female *n* = 5, male *n* = 5) and MNR (closed data points, female *n* = 6, male *n* = 5) pregnancies. Statistical analysis: two-way ANOVA (factors = diet and sex; Intx = interaction effect) with Tukey’s multiple comparison test. *P* < 0.05 was considered statistically significant. Data presented as mean ± SD

Multiple protein bands immunoreactive to anti-AR antibodies were detected in cytoplasmic and nuclear fractions of baboon placentae including AR-FL (110 kDa), AR-v (~ 80 kDa) and AR-45 (45 kDa; Fig. 4A). Cytoplasmic AR-45 expression was higher in male compared with female placentae independent of diet (sex effect [*F*_(1,15)_ = 42.74, *P* = 0.019], Fig. 4B) and was higher in MNR pregnancies (diet effect [*F*_(1, 15)_ = 18.47, *P* < 0.001], Fig. 4B), whereas cytoplasmic AR-v and AR-FL expression did not change by sex or diet (Fig. 4B). Nuclear AR-45 expression was higher in MNR pregnancies independent of sex (diet effect [*F*_(1, 16)_ = 6.637, *P* = 0.020], Fig. 4C), whereas nuclear AR-v and AR-FL expression did not differ by sex or diet (Fig. 4C).

### Placental ER protein expression is not impacted by maternal diet or sex

**Fig. 5:**
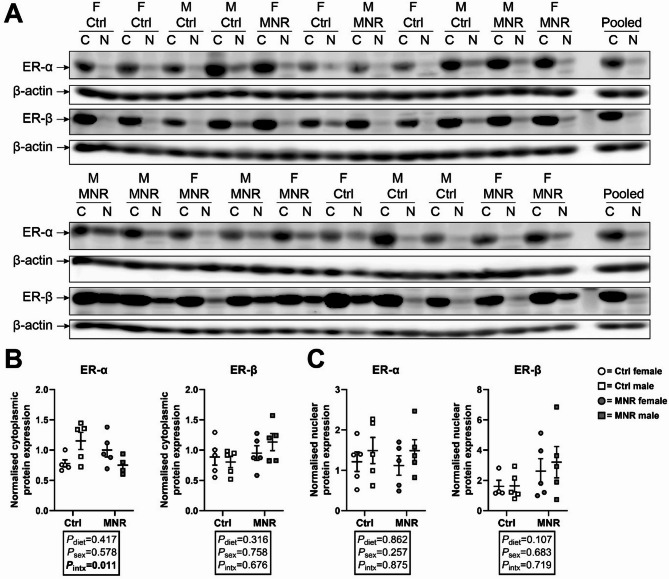
Estrogen receptor (ER) alpha and beta protein expression in the baboon placenta is impacted by maternal diet or sex. **(A)** Cytoplasmic (C) and nuclear (N) protein extracts of female (F) and male (M) placenta samples from control (Ctrl) and maternal nutrient restriction (MNR) pregnancies. ER-α and ER-β were detected in all samples. Membranes were probed with β-actin as a loading control. **(B)** Cytoplasmic and **(C)** nuclear expression of ER-α and ER-β in female (circles) and male (square) placentae of Ctrl (open data points, female *n* = 5, male *n* = 5) and MNR (closed data points, female *n* = 6, male *n* = 5) pregnancies. Statistical analysis: two-way ANOVA (factors = diet and sex; Intx = interaction effect) with Tukey’s multiple comparison test (*=*P* ≤ 0.05). *P* < 0.05 was considered statistically significant. Data presented as mean ± SD

ER-α and ER-β were detected in the cytoplasm and nucleus of baboon placentae (Fig. 5A), but expression did not differ by diet or sex (Fig. 5B & C).

### Gene expression of steroidogenic factors is impacted by placental sex but not maternal diet

**Fig. 6:**
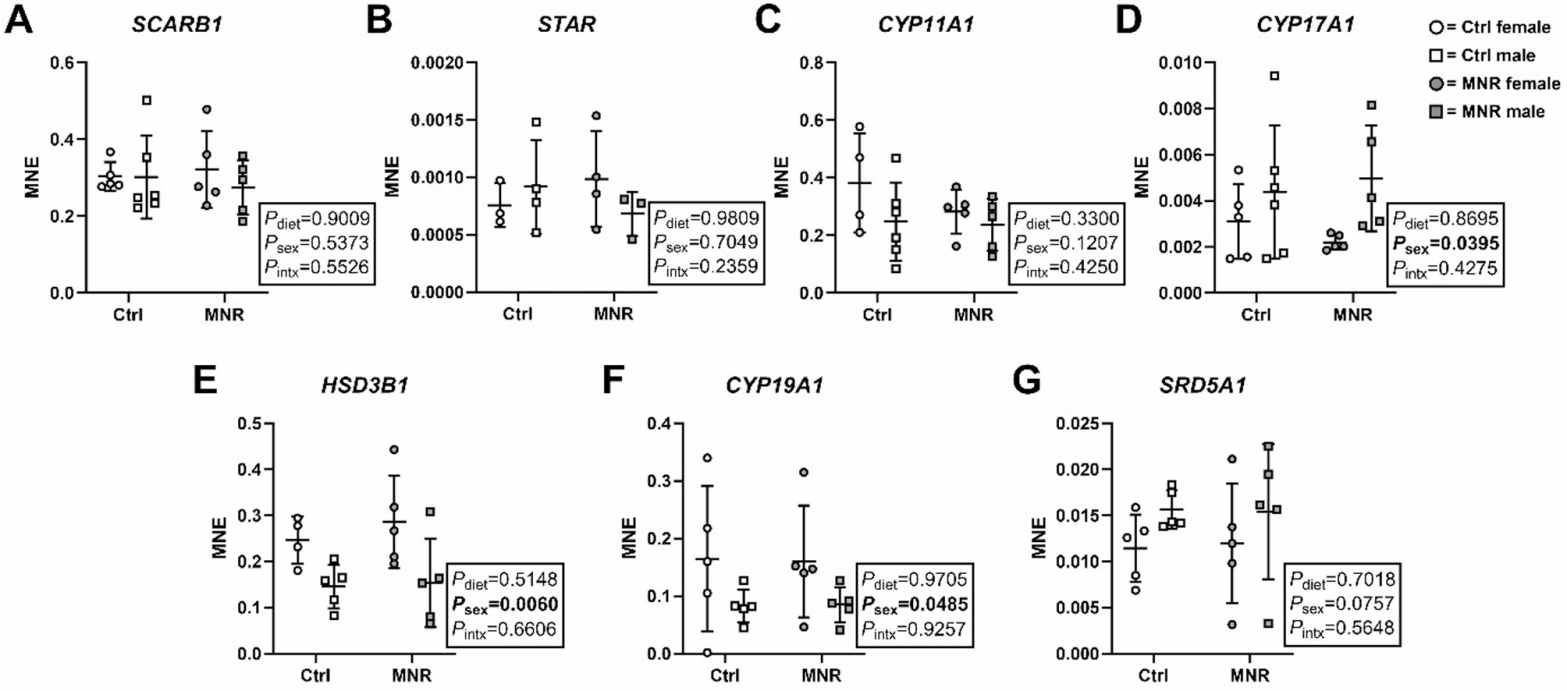
Expression of steroidogenic transcripts is impacted by sex but not maternal diet. Transcript expression of **(A)** *SCARB1*, **(B)** *STAR*, **(C)** *CYP11A1*, **(D)** *CYP17A1*, **(E)** *HSD3B1*, **(F)** *CYP19A1*, and **(G)** *SRD5A1* was measured in female (circle data points) and male (square data points) placenta samples from Control (Ctrl; open data points, female *n* = 5, male *n* = 6) and maternal nutrient restriction (MNR; closed data points, female *n* = 5, male *n* = 5) pregnancies. MNE = Mean normalised expression. Statistical analysis: two-way ANOVA (fixed factors: diet and sex; intx = interaction effect) with Tukey’s post hoc. *P* < 0.05 was considered statistically significant. Data presented as mean ± SD

*CYP17A1* expression was higher in males compared with females, independent of diet (sex effect [*F*_(1, 17)_ = 4.976, *P* = 0.0395], Fig. 6D), whereas *HSD3B1* and *CYP19A1* expression was lower in males compared with females, independent of diet (sex effect [*F*_(1, 15)_ = 10.20, *P* = 0.0060] and [*F*_(1, 16)_ = 4.560, *P* = 0.0485], respectively; Fig. 6E & F). *SCARB1*, *STAR*, and *SRD5A1* expression did not differ by maternal diet or sex (Fig. 6A, B, C & G).

## Discussion

To our knowledge, this is the first study to examine the effect of MNR and sex on placental steroidogenic and angiogenic profiles. Moreover, this study is the first to demonstrate that MNR impacts androgen signalling pathways in a sex-specific manner. The data herein partly support previous studies that suggest male placentae prioritise androgen-mediated signalling pathways compared with females. These data indicate that this evolutionary adaptation is enhanced in response to a maternal stressor, which may ensure fetal growth and masculinisation at the expense of placental function [37]. In contrast, females appear to dampen the androgen signalling pathway in response to MNR and enhance markers of angiogenesis when compared with males. Additionally, altered expression and subcellular localisation of AR protein variants in response to MNR suggests enhanced function of the antagonistic isoform, AR-45. However, the biological significance of this finding requires further investigation. Collectively, our data identified changes to biological processes required for normal placental growth and development are impacted by MNR and sex, which may have implications for sex-specific fetal growth and survival outcomes in MNR pregnancies.

The in vitro characterisation of testosterone-metabolising enzymes supports previous work demonstrating placental synthesis and metabolism of androgens [15]. Differences in the activity of these placental testosterone-metabolising enzymes may contribute to altered activity of the androgen signalling axis. We found that the activity of CYP3A7 – via testosterone to 2α-OHT metabolism – was higher in female compared with male MNR placentae. In humans, CYP3A7 is considered a fetal-specific CYP3A isoform, accounting for the majority of fetal CYP3A content [28], and plays an essential role in fetal androgen metabolism and homeostasis [25, 41]. Therefore, sex differences in CYP3A7 activity in response to MNR may have implications for the activity of androgen-mediated signalling pathways. While the biological function of 2α-OHT is not known, it is postulated that conversion of testosterone to less active ligands would result in reduced activity of the androgen signalling axis. Indeed, polycystic ovary syndrome (PCOS) patients carrying the CYP3A7*1 C variant – indicative of persistent expression of CYP3A7 through to adulthood – have lower androgen concentrations than PCOS noncarrier patients [18], which may overall dampen androgen signalling pathways.

We also found changes to aromatase (CYP19A1) activity and expression, which may further modulate placental-specific androgen signalling pathways and partly contribute to sex-specific differences in placental function in response to pregnancy complications. Placental aromatase function is well characterised, but no study has examined the impact of MNR on placental aromatase activity. In 21-day-old rats whose dams were fed either protein-energy restricted or energy-restricted diets, testis *Cyp19a1* expression was reduced [57]. Similarly, a maternal low-protein diet reduced aromatase mRNA expression but increased protein expression in the prepubertal gilt ovary [55]. Reduced placental aromatase expression has been implicated in placental dysfunction and may contribute to the pathogenesis of pregnancy-specific complications including preeclampsia [44]. Male-bearing pregnancies are at a greater risk of developing preeclampsia than female-bearing pregnancies [13]. Although the aetiology of preeclampsia is complex and involves dysregulation of multiple molecular pathways within the placenta, it is hypothesised that an imbalanced placental-specific androgen signalling axis may contribute to these risks [27]. We also reported reduced *CYP19A1* expression in male compared with female placentae, independent of diet. While these findings were not recapitulated in the in vitro activity studies, we did report a higher conversion rate of testosterone to estradiol in female MNR compared with female Control placentae. This sex-specific adaptation to a pregnancy stressor is consistent with previous work that demonstrated higher aromatase mRNA and protein expression in female placentae from preeclamptic pregnancies compared with sex-matched controls, whereas the opposite response was observed in males [49]. Collectively, our findings of altered aromatase expression and function support previous hypotheses that male placentae prioritise androgen-mediated pathways in response to maternal stressors whereas female placentae prioritise alternative pathways to support placental function.

Consistent with the above, the observed increase in *CYP17A1* and *SRD5A1*, alongside reduced *HSD3B1* and *CYP19A1* expression, in males compared with females further supports a male-specific preference for androgen synthesis and action in the placenta, which may impact placental function outcomes. Placental CYP17A1 metabolises pregnenolone and progesterone to the androgens dehydroepiandrosterone and androstenedione, respectively, whereas SRD5A1 converts testosterone to the more potent androgen ligand, DHT [35]. Increased placental *Cyp17a1* expression is associated with reduced fetal growth outcomes, particularly in females [47], whereas placental SRD5A1 protein expression is higher in male compared with female term placentae [60]. HSD3B1 metabolises pregnenolone to progesterone and is also involved in the production of estrogens: both classes of hormones are essential for maintained pregnancy health [31]. Our findings of reduced *HSD3B1* expression in males compared to females partly supports previous work in a maternal rat model of low protein diet, which found reduced placental *Hsd3b1* expression in protein restricted males compared with protein restricted females at 14- and 21-days gestation [16]. Reduced *HSD3B1* expression may exacerbate pregnancy risks: placental expression of *HSD3B1* is reduced in placentae from preeclamptic pregnancies [52], whereas extravillous trophoblasts have decreased *HSD3B1* expression in cases of recurrent early miscarriage [59]. Taken together, these results indicate sex differences in key steroidogenic genes, independent of maternal diet, may influence placental-specific androgen synthesis and thus modulate the androgen signalling axis. However, it is important to note that while we observed sex differences in *SRD5A1* expression, the activity of SRD5A1 did not differ between sexes.

Androgens are known to modulate key biological systems required for normal placental function. Increased androgen concentrations throughout pregnancy can reduce placental volume and weight [26], increase placental inflammatory pathways [56], and induce antiangiogenic responses that result in placental ischemia and fetal hypoxia [4, 19, 23]. Similar responses have been reported in pregnancies impacted by MNR [5]; however, it is not known whether these placental responses are the result of androgen-mediated mechanisms or alternative pathways impacted by MNR. Regardless, in the current study male placentae had decreased *ANGPT2*, but higher *PGF* expression, compared to female placentae, independent of diet. Distinct sexual dimorphism of *ANGPT2* and *PGF* expression may be indicative of poorer placental function in males. Tight regulation of angiopoietin-2 mediates placental vascular remodelling [12] and its dysfunction contributes to placental perturbations and hypoxia; in sheep and humans, late gestation placental expression of *ANGPT2* mRNA and protein is reduced in response to fetal growth restriction [12, 20]. Within the placenta, *PGF* expression is increased in a guinea pig model of early gestation maternal hypoxia [58], but is also reduced in response to human fetal growth restriction [61]. Considering we did not report any sex differences in placental weight or fetal body weight, nor the fetal-placental weight ratio, it may be argued that the increased expression of *PGF* in males is partly explained by the concomitant decrease in *ANGPT2* expression; however, the physiological implications of these findings require further investigation. We also found higher *KDR* expression in female MNR compared with female Control, which may partly be explained by the increase in CYP19A1 activity; previous work identified *KDR* expression is upregulated in an estrogen-dependent manner [17, 21].

Although we did not find changes in the expression or subcellular localisation of ER proteins, cytoplasmic expression of the antagonistic AR isoform, AR-45, was higher in male compared with female placentae, and both cytoplasmic and nuclear AR-45 expression was increased in response to MNR. Placental AR-45 expression has previously been reported in humans and sheep [36, 38], with nuclear expression increased in response to maternal stressors, particularly in males. In the current study, higher cytoplasmic AR-45 expression in males may suggest reduced nuclear translocation, thereby enhancing AR-FL action within the nucleus [30]. This is despite no change observed in AR-FL cytoplasmic or nuclear expression. In contrast, higher AR-45 expression in both the cytoplasm and nucleus in response to MNR may offer beneficial, antiandrogenic properties. Indeed, gene therapy with AR-45 in a mouse model of spinal-bulbar muscular atrophy (associated with dysregulated androgen signalling) restored phenotype via altered AR transcriptional activity [30]. It is tempting to speculate that the observed increase in AR-45 expression may be an attempted adaptation to modulate the androgen signalling axis in response to perturbations induced by MNR, such as an increase in androgen ligands. However, further work is required to define the role of AR-45 in the placenta and determine whether sex-specific changes to isoform-specific downstream pathways are impacted by complications of pregnancy such as MNR.

While non-human primates are excellent models of human health and disease, they are a highly resource-demanding model to establish and maintain [22]. This limits the number of animals included in study groups; it would therefore benefit future works to expand on the current findings using larger cohorts in this or other species, but this is beyond the current study’s scope and feasibility. In addition, while our study effectively models a 30% global nutrient restriction, it is recognised that this does not recapitulate the heterogeneity of nutrient deficits during human pregnancy. Another limitation is that we were unable to quantify maternal or fetal concentrations of androgens throughout pregnancy, which is in part due to the inability to chronically catheterise the non-human primate maternal and fetal systems to collect blood samples across gestation. Despite our study not identifying differences in the expression of glucose transporters or growth factors, additional pathways involved in fetoplacental growth that are known to be perturbed by androgen signalling (i.e. cellular respiration [42] and amino acid transportation [48]) may be impacted by MNR in a sex-specific manner. Evidently, future studies that combine comprehensive in vivo measures and molecular profiling are required to untangle the complexity of the placental-specific androgen signalling axis, define how pregnancy stressors associated with excess maternal androgens impact the function of this signalling axis, and characterise whether observed responses are impacted by placental sex.

## Conclusion

Overall, our findings demonstrate sex-specific differences in the expression and function of factors involved in androgen metabolism and signalling. Specifically, we show male placentae prioritise androgen-dependent signalling pathways, independent of a maternal stressor induced by MNR, whereas the female-specific response to MNR may enhance placental function. The observed sex-specific placental adaptations, or lack thereof, to intrauterine stressors associated with MNR further supports the ‘male disadvantage’ hypothesis. Importantly, our findings highlight a need for future, sex-informed studies that assess the efficacy and safety of novel therapeutics that modulate placental-specific androgen signalling throughout pregnancy. By doing so, placental function may be improved in complicated pregnancies associated with increased androgen concentrations.

## Data Availability

All data supporting the results are presented in the manuscript.
